# Pooled prevalence and associated factors of teenage pregnancy among women aged 15 to 19 years in sub-Saharan Africa: evidence from 2019 to 2022 demographic and health survey data

**DOI:** 10.1186/s40834-024-00289-5

**Published:** 2024-05-23

**Authors:** Enyew Getaneh Mekonen

**Affiliations:** Department of Surgical Nursing, School of Nursing, College of Medicine and Health Sciences, University of Gondar, Gondar, Ethiopia

**Keywords:** Teenage pregnancy, Teenagers, DHS, Sub-Saharan Africa, Multilevel analysis

## Abstract

**Background:**

Teenage pregnancy is becoming one of the most common social and public health problems worldwide, with the highest prevalence in sub-Saharan Africa. Health risks and adverse outcomes of pregnancy and childbirth among adolescent girls are the commonest cause of the global burden of maternal morbidity and mortality. This study is intended to determine the pooled prevalence and determinants of teenage pregnancy in sub-Saharan Africa using the most recent demographic and health survey data (2019–2022).

**Methods:**

A cross-sectional study was conducted using data from the most recent demographic and health surveys of four countries (Kenya, Tanzania, Gabon, and Cameroon) in sub-Saharan Africa conducted between 2019 and 2022. A total weighted sample of 12,829 teenagers aged 15 to 19 years was included in the study. Data extracted from demographic and health survey data sets were cleaned, recorded, and analyzed using STATA/SE version 14.0 statistical software. Multilevel mixed-effects logistic regression was used to determine the factors associated with the dependent variable. Finally, variables with a *p*-value ≤ 0.05 and an adjusted odds ratio with a 95% confidence interval were declared statistically significant.

**Results:**

The pooled prevalence of teenage pregnancy among women aged 15 to 19 years was 18.15% (95% CI: 17.49, 18.83). Teenage pregnancy was positively associated with the respondent’s age [AOR = 2.97; 95% CI (2.55, 3.46)], educational status [AOR = 2.21; 95% CI (1.62, 3.03)] and [AOR = 1.80; 95% CI (1.54, 2.12)], wealth status [AOR = 2.61; 95% CI (2.12, 3.22)] and [AOR = 1.65; 95% CI (1.33, 2.05)], relation to the household head [AOR = 2.09; 95% CI (1.60, 2.72)], and unmet need for contraception [AOR = 14.3; 95% CI (11.5, 17.8)]. On the other hand, it was negatively associated with marital status [AOR = 0.08; 95% (0.07, 0.10)], working status [AOR = 0.75; 95% CI (0.64, 0.88)], age at first sex [AOR = 0.68; 95% CI (0.58, 0.80)], contraceptive use [AOR = 0.25; 95% CI (0.20, 0.30)], contraceptive knowledge [AOR = 0.27; 95% CI (0.19, 0.40)], and community contraceptive utilization [AOR = 0.85; 95% CI (0.73, 0.99)].

**Conclusion:**

In the current study, one out of six young women aged 15 to 19 experienced teenage pregnancy. Therefore, addressing unmet needs for family planning, improving women’s educational status, and giving special attention to teenagers with low educational and economic status are recommended.

## Background

Teenage pregnancy is becoming one of the most common social and public health problems worldwide, with a fluctuating prevalence rate [1]. An estimated 21 million girls aged 15 to 19 had pregnancies each year in low- and middle-income countries (LMICs), of which around half were unplanned and which resulted in approximately 12 million births [2, 3]. More than half of unplanned pregnancies among girls aged 15 to 19 end in unsafe abortions in LMICs [3]. Despite the decline in adolescent birth rates worldwide, it is reported that over 100 births per 1,000 women occur in sub-Saharan Africa (SSA), with an estimated actual number of 6,114,000 births among girls aged 15–19 years [4]. Teenage pregnancy is associated with maternal and child mortality, as well as other severe neonatal complications, and has a negative impact on the mental and social wellbeing of teenagers [5, 6].

Around 15% of the global burden of maternal morbidity is attributed to health risks during pregnancy and childbirth among adolescent girls [7]. Adverse outcomes of pregnancy and childbirth are the second leading cause of mortality for girls aged 15–19 years in the world [8]. Teenage pregnancy is associated with both adverse maternal and neonatal outcomes [9]. Preeclampsia (a progressive hypertensive disorder of pregnancy with multi-organ involvement) that leads to adverse maternal and perinatal consequences is common among teen mothers [10, 11]. Adolescent girls are also more prone to preterm premature rupture of membranes as they have immature uterine and cervical blood circulation, making them more susceptible to underdiagnosed or diagnosed infections [12]. As higher iron intake is vital for a particular state of rapid growth where major biological adjustments are in process, pregnant teenagers have a greater risk of anemia, which ends up with physical and cognitive damage to both mothers and fetuses [13]. Lack of early sex education, substance abuse, gender inequality, and false beliefs increase the risk of sexually transmitted diseases among teenagers [14].

Preterm birth, low birth weight, low Apgar score, stillbirths, and neonatal mortality are the neonatal outcomes of teenage pregnancy [9]. Preterm birth in teen women could be associated with a low number of prenatal visits, a late onset of prenatal care, and a low educational level [15]. Non-acceptance of pregnancy, fewer prenatal consultations, not having standardized nutritional care, and preterm delivery are the factors that lead to low birth weight in teenage pregnancy [16]. A low Apgar score is a more predominant outcome of teenage pregnancy than adults as a result of different socio-demographic, nutritional, and obstetric factors [17]. The biological immaturity of teenagers who are still developing can trigger fetal-maternal competition for nutrients as the pregnancy progresses, hence threatening fetal growth, development, and survival as the pregnancy proceeds, which leads to stillbirths [18]. Teenage pregnancy is also associated with neonatal mortality mediated through preterm delivery and neonates being small for gestational age [19].

Psychological concerns (suicidal thoughts, guilt, loneliness, worry, stress), difficulties returning to school, low socioeconomic level, social stigmatization, postpartum depression, developmental delays, and behavioral issues later in life are long-term consequences of teenage pregnancy [20, 21]. Preventing pregnancy among teenagers and pregnancy-related morbidity and mortality are basic to achieving positive health outcomes across the life course and vital for achieving the sustainable development goals related to maternal and newborn health [22]. Previous studies conducted elsewhere showed that the prevalence of teenage pregnancy was 44.3% in sub-Saharan Africa [23], 24.88% in sub-Saharan Africa high fertility countries [24], 13.42% in the Gambia [25], and 54.6% in East Africa [26]. Studies also showed that age, contraceptive utilization, family size, knowledge about contraceptives, marital status, age at first marriage, working status, household wealth status, community-level contraceptive utilization, residence, media exposure, unmet need for family planning, educational level, feelings about the current pregnancy, and relation to the household head were significantly associated with teenage pregnancy [23–30].

Even though there are studies conducted on the magnitude and determinants of teenage pregnancy in east Africa and SSA [23, 24, 26], there is no evidence on the pooled prevalence and determinants of teenage pregnancy in the region conducted using the most recent demographic and health surveys (DHS) dataset. Therefore, this study is intended to determine the pooled prevalence and determinants of teenage pregnancy in SSA using the most recent DHS data (2019–2022). Thus, the findings of the current study could help program managers, policymakers, and non-governmental organizations work on maternal and child health to plan programs and interventions for teenage pregnancy and related adverse health outcomes.

## Methods and materials

### Data sources, study design, and sampling

A cross-sectional pooled dataset using the most recent DHS from four SSA countries, which was conducted between 2019 and 2022, was employed. Demographic and health surveys from four sub-Saharan African countries, including Kenya (2022), Tanzania (2022), Gabon (2019–21), and Cameroon (2022), were used. The data were appended to figure out the pooled prevalence of teenage pregnancy and its associated factors in SSA countries. Different datasets, including those for children, males, women, births, and households, are included in the DHS for each country. For this study, the individual record (IR) file was used. The DHS is a nationwide survey, mostly collected every five years across LMICs. It makes cross-country comparison possible as it uses standard procedures for sampling, questionnaires, data collection, cleaning, coding, and analysis [31].

A total weighted sample of 12,829 teenagers aged 15 to 19 years was included in the study (Table 1). The DHS employs a stratified, two-stage sampling technique [32]. The first stage involves the development of a sampling frame, consisting of a list of primary sampling units (PSUs) or enumeration areas (EAs), which covers the entire country and is usually developed from the latest available national census. The second stage is the systematic sampling of households listed in each cluster, or EA. Further information on the survey sampling strategies is available in the DHS guideline [33].

**Table 1:** Sample size for teenage pregnancy and its associated factors among women aged 15 to 19 years in sub-Saharan African countries

Country	Year of survey	Weighted sample (*n*)	Weighted sample (%)
Cameroon	2022	1,394	10.87
Gabon	2019-21	1,889	14.72
Kenya	2022	6,404	49.92
Tanzania	2022	3,142	24.49
Total sample size		12,829	100

### Variables of the study

#### Outcome variable

Teenage pregnancy (yes, no): a woman was considered to be experiencing teenage pregnancy if her age was from 15 to 19, she had had a birth, and she had not had a birth but was pregnant at the time of the interview [33].

#### Explanatory variables

Individual-level factors: age of respondents (15–17 years, 18–19 years), marital status (unmarried, married), age at first sex (less than 16 years, 16–19 years), contraceptive use (no, yes), educational status (no education, primary, secondary/higher), occupation (working, not working), household wealth status (poor, middle, rich), sex of the household head (male, female), relation to household head (head/spouse, daughter, relative/other), exposure to media (no, yes), knowledge of contraceptives (knows no methods, knows traditional/modern methods), unmet need for contraception (no, yes).

Community-level factors: community education level (low, high), community poverty level (low, high), community contraceptive utilization (low, high), community media exposure (low, high), and residence (urban, rural).

### Operational definitions

#### Media exposure

Generated by combining whether a respondent reads newspapers or magazines, listens to the radio, or watches television, and is coded as “yes” if the mother was exposed to at least one of these media and “no” otherwise.

#### Community media exposure

The proportion of women who had been exposed to at least one media (television, radio, or newspaper) and categorized based on the national median value as low (communities with ≤ 50% of women exposed) and high (communities with > 50% of women exposed).

#### Community education level

The proportion of women with a minimum primary level of education derived from data on respondents’ level of education. Then, it was categorized using the national median value into two categories: low (communities with ≤ 50% of women having at least primary education) and high (communities with > 50% of women having at least primary education).

#### Community contraceptive utilization

Measured by the proportion of women who used any or a combination of the contraceptives. It was categorized based on national media value as low (community in which ≤ 50% of women used any contraceptive methods) and high (community with > 50% of women using any contraceptive methods).

#### Community poverty level

An aggregated variable from household wealth status (proportion of women from poor and rich wealth status), and it was recoded as low and high community poverty level, likewise.

### Data management and analysis

Data extracted from the most recent DHS data sets were cleaned, recorded, and analyzed using STATA/SE version 14.0 statistical software. Sample weight was employed to manage sampling errors and non-responses. Continuous variables were categorized, and categorical variables were further re-categorized. Descriptive analysis was carried out to present the data in frequencies and percentages. Both the individual and community-level variables were presented using descriptive statistics. The DHS data’s variables were organized in clusters; 12,829 women are nested within households, and households were nested within 1692 clusters. The assumptions of independent observations and equal variance across clusters were broken to employ the traditional logistic regression model. This is an indication that using a sophisticated model to take into account between-cluster factors is necessary. As a result, multilevel mixed-effects logistic regression was used to determine the factors associated with teenage pregnancy. Multilevel mixed effect logistic regression follows four models: the null model (outcome variable only), model I (only individual-level variables), model II (only community-level variables), and model III (both individual and community-level variables). The model without independent variables (the null model) was used to check the variability of teenage pregnancy across the cluster. The association of individual-level variables with the outcome variable (Model I) and the association of community-level variables with the outcome variable (Model II) were assessed. In the final model (Model III), the association of both individual and community-level variables was fitted simultaneously with the outcome variable (teenage pregnancy).

The magnitude of the clustering effect and the degree to which community-level factors explain the unexplained variance of the null model were quantified by checking the intra-class correlation coefficient (ICC) and proportional change in variance (PCV). A model with the lowest deviance was selected as the best-fitted model. Finally, variables with a *p*-value less than 0.05 and an adjusted odds ratio (AOR) with a 95% confidence interval (CI) were described as statistically significant variables associated with teenage pregnancy. The presence of multi-collinearity between covariates was checked by using a variance inflation factor (VIF) falling within acceptable limits of 1–10, indicating the absence of significant collinearity across independent variables.

### Random effect model

Random effects or measures of variation of the outcome variable were estimated using the median odds ratio (MOR), ICC, and PCV. The variation between clusters was measured by the ICC and PCV. Taking clusters as a random variable, the ICC reveals that the variation of teenage pregnancy between clusters is computed as ICC = VC/(VC + 3.29) ×100%. The MOR is the median value of the odds ratio between the area of the highest risk and the area of the lowest risk for teenage pregnancy when two clusters are randomly selected, using clusters as a random variable; MOR = 𝑒 0.95√VC. In addition, the PCV demonstrates the variation in the prevalence of teenage pregnancy explained by factors and computed as: PCV = (Vnull-VC)/Vnull×100%, where Vnull = variance of the null model and VC = cluster level variance [34]. The fixed effects were used to estimate the association between the likelihood of teenage pregnancy and individual and community-level independent variables.

## Results

### Individual- and community-level characteristics of study subjects

A total of 12,829 young women aged 15 to 19 years were included in this study. The mean age of the women was 16.96 ± 0.01 years, and 60.85% of them fall in the age range of 15–17 years. More than half (62.80%) of the women completed secondary or higher education, and 88.74% of them were unmarried. Regarding working status, 80.23% of women had no work, and more than three-fourths (77.85%) of them had media exposure. More than one-third (34.98%) of the women in SSA had rich socioeconomic status. The majority (90.24%) of women didn’t use contraceptives, and 80.94% of them were aged less than 16 years at their first sex. The household head was male for 64.48% of the women, and their relation to the household head was daughter for 72.43% of them. The majority (91.60%) of the women knew traditional or modern methods of contraception, and only 5.79% of them had an unmet need for contraception. More than half (58.38%) of the study subjects were from rural areas, and 44.54% of them had low community-level media exposure. More than half (51.07%) of the women had low community-level poverty, and 68.47% of them had high community-level education. High community contraceptive utilization was reported among 39.28% of the women aged 15 to 19 years (Table 2).

**Table 2:** Individual-and community-level characteristics of study subjects, pooled data from four SSA countries, DHS 2019–2022 (*n* = 12,829)

Variables	Category	Frequency (*n*)	Percentage (%)
Age of respondents	15–17 years	7,807	60.85
	18–19 years	5,022	39.15
Educational status	No formal education	789	6.15
	Primary	3,983	31.05
	Secondary and higher	8,057	62.80
Marital status	Married	1,288	11.26
	Unmarried	10,147	88.74
Occupation	Not working	9,172	80.23
	Working	2,260	19.77
Exposure to media	Yes	9,988	77.85
	No	2,841	22.15
Household wealth status	Poor	5,703	44.45
	Middle	2,639	20.57
	Rich	4,487	34.98
Age at first sex	Less than 16 years	9,255	80.94
	16–19 years	2,180	19.06
Contraceptive use	No	10,319	90.24
	Yes	1,116	9.76
Sex of the household head	Male	8,272	64.48
	Female	4,557	35.52
Relation to household head	Head/spouse	1,031	8.04
	Relative or other	2,506	19.53
	Daughter	9,292	72.43
Knowledge of contraceptives	Knows no methods	961	8.40
	Knows traditional/modern methods	10,474	91.60
Unmet need for contraception	No	12,086	94.21
	Yes	743	5.79
Residence	Urban	5,339	41.62
	Rural	7,490	58.38
Community media exposure	Low	5,714	44.54
	High	7,115	55.46
Community poverty level	Low	6,552	51.07
	High	6,277	48.93
Community education level	Low	4,045	31.53
	High	8,784	68.47
Community contraceptive utilization	Low	7,788	60.72
	High	5,038	39.28

### Pooled prevalence of teenage pregnancy

**Fig. 1:**
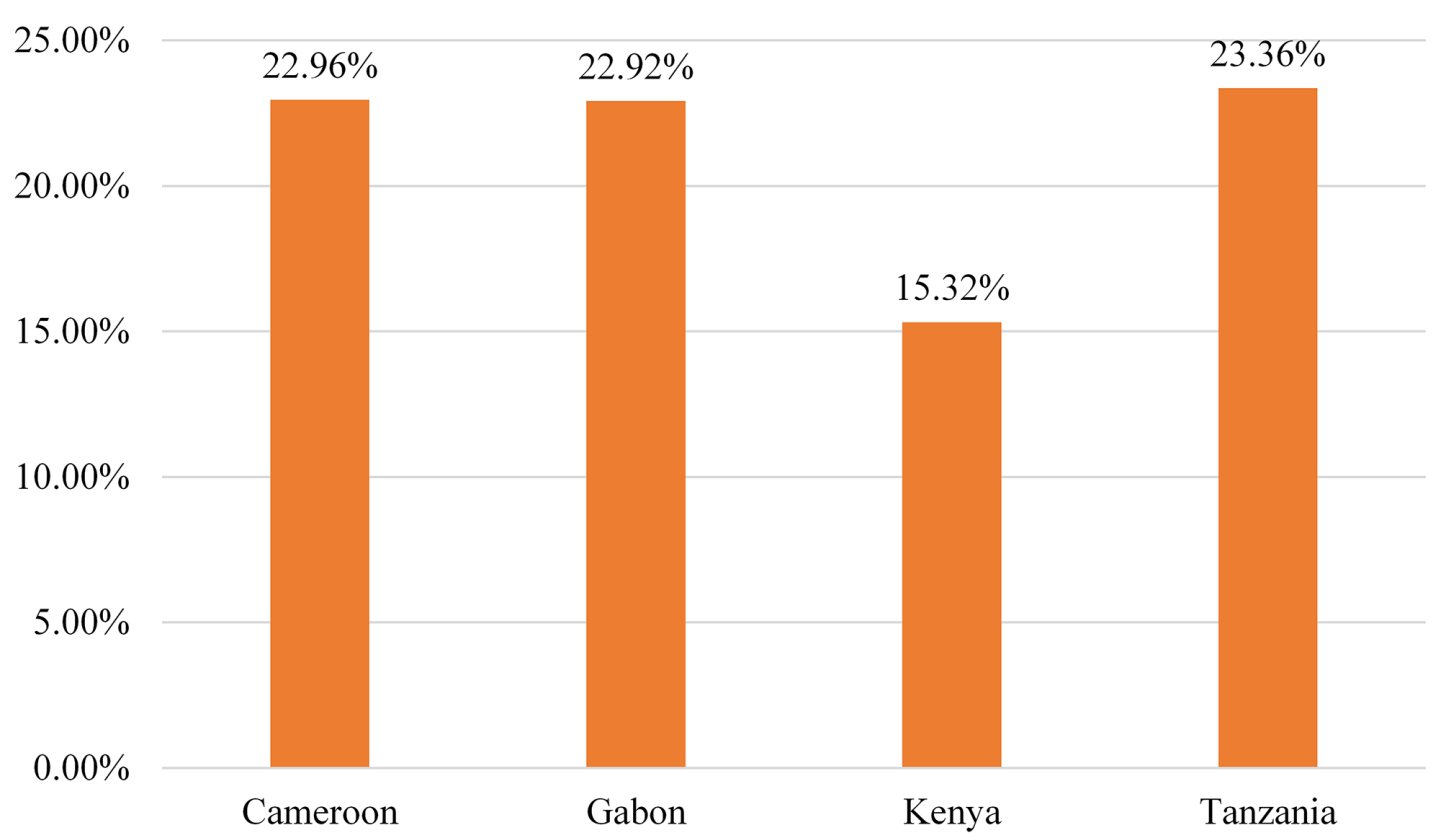
Prevalence of teenage pregnancy in four SSA countries, DHS 2019–2022 (*n* = 12,829)

In the current study, the pooled prevalence of teenage pregnancy among women aged 15 to 19 years was 18.15% (95% CI: 17.49, 18.83). The highest prevalence of teenage pregnancy was reported in Tanzania (23.36%) and the lowest in Kenya (15.32%) (Fig. 1).

### Measures of variation and model fitness

A null model was used to determine whether the data supported the decision to assess randomness at the community level. Findings from the null model showed that there were significant differences in teenage pregnancy between communities, with a variance of 0.224 and a *P* value of < 0.001. The variance within clusters contributed 93.63% of the variation in teenage pregnancy, while the variance across clusters was responsible for 6.37% of the variation. In the null model, the odds of teenage pregnancy differed between higher and lower-risk clusters by a factor of 1.45 times. The intra-class correlation value for Model I indicated that 4.38% of the variation in teenage pregnancy accounts for the disparities between communities. Then, with the null model, we used community-level variables to generate Model II. According to the ICC value from Model II, cluster variations were the basis for 3.47% of the differences in teenage pregnancy. In the final model (model III), which attributed approximately 4.36% of the variation in the likelihood of teenage pregnancy to both individual and community-level variables, the likelihood of teenage pregnancy varied by 1.44 times across low and high teenage pregnancies. A model with the lowest deviance (-2LLR) was selected as the most suitable model for the data (model III) (Table 3).

**Table 3:** Model comparison and random effect analysis for teenage pregnancy and its associated factors in Sub-Saharan African countries, DHS 2019–2022 (*n* = 12,829)

Parameter	Null model	Model I	Model II	Model III
Variance	0.223998	0.1506988	0.1183353	0.1499781
ICC	6.37%	4.38%	3.47%	4.36%
MOR	1.57	1.45	1.39	1.44
PCV	Reference	32.72%	47.17%	33.04%
**Model fitness**				
LLR	-6050.558	-3078.3159	-5916.6495	-3074.9047
Deviance	12,101.116	6,156.6318	11,833.299	6,149.8094

ICC: Intra cluster correlation, LLR: log-likelihood ratio, MOR: median odds ratio, PCV: Proportional change in variance

### Individual- and community-level factors associated with teenage pregnancy

In the final fitted model of multivariable multilevel logistic regression analysis, age of respondents, educational level, marital status, occupation, wealth index, age at first sex, contraceptive use, relation to household head, knowledge of contraceptives, unmet need for contraception, and community contraceptive utilization were factors significantly associated with teenage pregnancy.

The odds of teenage pregnancy were nearly three times higher among women aged 18–19 years compared with those aged 15–17 years [AOR = 2.97; 95% CI (2.55, 3.46)]. Women who had no formal education and completed primary education were 2.21 and 1.80 times more likely to have teenage pregnancy than women who completed secondary education or higher, respectively [AOR = 2.21; 95% CI (1.62, 3.03)] and [AOR = 1.80; 95% CI (1.54, 2.12)]. Married women were 99.92% less likely to experience teenage pregnancy than unmarried women [AOR = 0.08; 95% (0.07, 0.10)]. Non-working women were 25% less likely to have teenage pregnancies compared with their counterparts [AOR = 0.75; 95% CI (0.64, 0.88)]. Household wealth status was another factor significantly associated with teenage pregnancy. Women with poor and middle wealth status were 2.61 and 1.65 times more likely to experience teenage pregnancy than those with rich wealth status, respectively [AOR = 2.61; 95% CI (2.12, 3.22)] and [AOR = 1.65; 95% CI (1.33, 2.05)].

Similarly, women aged less than 16 years at their first sex were 32% less likely to experience teenage pregnancy than those aged 16–19 years [AOR = 0.68; 95% CI (0.58, 0.80)]. Teenagers who didn’t utilize contraceptives were 75% less likely to experience pregnancy compared with their counterparts [AOR = 0.25; 95% CI (0.20, 0.30)]. The odds of teenage pregnancy were 2.09 times higher among head/spouse women compared with daughters [AOR = 2.09; 95% CI (1.60, 2.72)]. Women who knew no methods of contraception were 73% less likely to have teenage pregnancies than those who knew traditional or modern methods [AOR = 0.27; 95% CI (0.19, 0.40)]. The odds of teenage pregnancy were 14.3 times higher among teenagers with an unmet need for contraception compared with their counterparts [AOR = 14.3; 95% CI (11.5, 17.8)]. Finally, teenagers from communities with low contraceptive utilization were 15% less likely to experience teenage pregnancy than those from communities with high contraceptive utilization [AOR = 0.85; 95% CI (0.73, 0.99)] (Table 4).

**Table 4:** Multivariable multilevel logistic regression analysis of individual and community-level factors associated with teenage pregnancy in SSA countries, DHS 2019–2022 (*n* = 12,829)

Variables	Category	Model I AOR (95% CI)	Model II AOR (95% CI)	Model III AOR (95% CI)
Age	18–19 years	2.96 (2.57, 3.44)*		2.97 (2.55, 3.46)*
	15–17 years	1		1
Educational status	No formal education	2.15 (1.59, 2.91)*		2.21 (1.62, 3.03)*
	Primary	1.78 (1.52, 2.08)*		1.80 (1.54, 2.12)*
	Secondary and above	1		1
Marital status	Married	0.08 (0.07, 0.11)*		0.08 (0.07, 0.10)*
	unmarried	1		1
Occupation	Not working	0.75 (0.65, 0.88)*		0.75 (0.64, 0.88)*
	Working	1		1
Exposure to media	No	0.98 (0.83, 1.17)		0.98 (0.82, 1.18)
	Yes	1		1
Wealth index	Poor	2.39 (2.00, 2.85)*		2.61 (2.12, 3.22)*
	Middle	1.58 (1.28, 1.94)*		1.65 (1.33, 2.05)*
	Rich	1		1
Age at first sex	Less than 16 years	0.68 (0.58, 0.80)*		0.68 (0.58, 0.80)*
	16–19 years	1		1
Contraceptive use	No	0.23 (0.19, 0.27)*		0.25 (0.20, 0.30)*
	Yes	1		1
Sex of the household head	Male	0.94 (0.82, 1.08)		0.95 (0.82, 1.09)
	Female	1		1
Relation to household head	Head/spouse	2.13 (1.64, 2.78)*		2.09 (1.60, 2.72)*
	Relative or other	0.93 (0.77, 1.12)		0.92 (0.77, 1.11)
	Daughter	1		1
Knowledge of contraceptives	Knows no methods	0.27 (0.18, 0.40)*		0.27 (0.19, 0.40)*
	Knows traditional/modern methods	1		1
Unmet need for contraception	Yes	14.7 (11.8, 18.2)*		14.3 (11.5, 17.8)*
	No	1		1
Residence	Rural		1.32 (1.18, 1.46)*	0.92 (0.78, 1.09)
	Urban		1	1
Community media exposure	Low		1.21 (1.07, 1.36)*	1.03 (0.87, 1.23)
	High		1	1
Community poverty level	High		1.20 (1.07, 1.34)*	0.90 (0.75, 1.06)
	Low		1	1
Community education level	Low		1.53 (1.36, 1.73)*	0.99 (0.82, 1.19)
	High		1	1
Community contraceptive utilization	Low		0.57 (0.52, 0.64)*	0.85 (0.73, 0.99)*
	High		1	1

*Statistically significant at *p*-value < 0.05

## Discussion

The current study aims to determine the pooled prevalence and associated factors of teenage pregnancy among women aged 15 to 19 years using the most recent DHS data in SSA countries. In this study, the pooled prevalence of teenage pregnancy was 18.15% (95% CI: 17.49, 18.83). This finding was lower than studies conducted in East Africa (54.6%) [26], Uganda (20.6% and 35.8%) [29, 35], five African countries (25.4%) [36], high-fertility sub-Saharan Africa (24.9%) [24], Nigeria (19%) [27], Tanzania (29%) [37], Zambia (29.9%) [38], and Nepal (29.1%) [39]. On the other hand, this finding was higher than studies conducted in Gambia (13.4%) [25]. This discrepancy might be due to socio-cultural, study period, study setting, and sample size differences like marital age, age at first sex, and contraceptive utilization. Most of the previous studies were conducted in a single area or single country, whereas the current study uses pooled data from four countries. The increased awareness about the impact of teenage pregnancy might also be the reason, as this study uses the most recent DHS data. The difference might also be explained by differences in the number of teenagers included in the study and the topographical distribution of adolescents.

This study also identified individual and community-level factors associated with teenage pregnancy. Accordingly, the odds of teenage pregnancy were nearly three times higher among women aged 18–19 years compared with those aged 15–17 years. Similar findings were reported by studies conducted in east Africa [26], sub-Saharan Africa [23], five African countries [36], Nigeria [27, 40], and Indonesia [30]. This might be due to the fact that as the age of teenagers increases, the probability of being involved in sexual intercourse and getting married will also increase. This will result in an increased chance of getting pregnant and childbearing. Women with low educational levels were more likely to have teenage pregnancies. This finding was in agreement with studies conducted in east Africa [26], Uganda [29, 35], Nigeria [27], Tanzania [37], sub-Saharan Africa [23], high-fertility sub-Saharan Africa [24], and Nepal [39]. This might be attributed to the fact that adolescent girls who had advanced education or who persisted in school longer are less likely to experience teenage pregnancy than adolescent girls who had no education [41]. As the educational status of teenagers increases, their chances of getting information about contraceptives and the negative impact of teenage pregnancy on their health will increase. Similarly, unmarried women were more likely to experience teenage pregnancy than married women. This finding was in contrast to studies conducted in east Africa [26], high-fertility sub-Saharan Africa [24], sub-Saharan Africa [23], and Nigeria [27]. This might be due to an adolescent girl’s desire to have children before she gets married. Unmarried teenagers may also be exposed to recurrent and unsafe sexual activity, which leads to an early pregnancy. The lack of support from their husbands to use family planning may be another possible reason.

Occupation was another significant factor in which non-working women were less likely to have teenage pregnancies compared with their counterparts. This finding was consistent with studies conducted in east Africa [26] and sub-Saharan Africa [23]. This may be explained by the fact that young women are exposed to different risky sexual practices at work, which might result in sexual assault and subsequent pregnancy [42]. Women with poor and middle wealth status were more likely to experience teenage pregnancy than those with rich wealth status, respectively. This finding was consistent with studies conducted in east Africa [26], sub-Saharan Africa [23], high-fertility sub-Saharan Africa [24], Gambia [25], and Tanzania [37]. This might be due to early exposure to marriage and sexual initiation, an inability to afford the cost of contraceptives, running away from homes looking for jobs, and early sexual practices to fulfill their basic needs that increase the risk of pregnancy. Women aged less than 16 years at their first sex were less likely to experience teenage pregnancy than those aged 16–19 years. This finding was in contrast with studies conducted in east Africa [26] and sub-Saharan Africa [23]. This could be explained by the effects of unsafe sex and contraceptive non-utilization to prevent teenage pregnancy among teenagers with late sexual initiation.

The odds of teenage pregnancy were two times higher among head/spouse women compared with daughters. This finding was supported by studies conducted in East Africa [26] and five East African countries [43]. This might be justified by the exposure of teenage girls to early sexual commencement, pregnancy, and early maternity due to the lack of parental support and supervision as they didn’t live with both of their genetic parents [43]. Knowing traditional or modern methods of contraception increases the odds of teenage pregnancy. This finding was in agreement with a study conducted in sub-Saharan Africa [23]. If there were no external incentives to postpone having children, the desire or social pressure to become pregnant could have led to pregnancy even when the individual knew about contraception [44]. Another significant barrier to the use of contraceptives among teens is the presence of societal standards that criticize early sexual involvement, pregnancy, and the use of contraception [45]. Furthermore, information about contraception might be inaccurate and filled with misconceptions, particularly if it comes from questionable sources as opposed to trustworthy ones [46]. Even among teenagers who are well-informed about contraception, stigma and prejudice from healthcare professionals, as well as anxiety about adverse effects, frequently prevent adolescents from getting and utilizing contraceptives [47]. The odds of teenage pregnancy were higher among teenagers with an unmet need for contraception. A study conducted in high-fertility sub-Saharan Africa [24] reported a similar finding. However, this finding was inconsistent with a study conducted in sub-Saharan Africa [23]. This might be due to the unmet need for contraception, which exposes teenagers to the risk of an unplanned pregnancy [48]. Thus, it is better to resolve the unmet need for contraception among teenagers to reduce teenage pregnancy in the study settings. Furthermore, teenagers from a community with high contraceptive utilization were more likely to experience teenage pregnancy. Likewise, teenagers who use contraceptives were more likely to experience pregnancy compared with their counterparts. This finding was consistent with a study conducted in East Africa [26]. This may be connected to the fact that, despite rising contraceptive use in poor nations, contraceptive failure, which leads to unintended and undesired pregnancy, remains widespread due to insufficient contraceptive counseling, awareness, and utilization skills [49]. Contraceptive needs, such as inconsistent usage and supply disruption, may still not be addressed, as seen by the increased prevalence of teenage pregnancy among users of contraception [50].

### Strengths and limitations of the study

Firstly, the study uses a large sample size from weighted nationwide representative data from four sub-Saharan African countries. Secondly, to get a reliable standard error and estimate by accommodating the hierarchical nature of the DHS data, the study uses a multilevel analysis. Thirdly, this finding can give program managers an insight into how to design and implement suitable intervention approaches since it uses national survey data. The study also has some limitations. There might be a possibility of recall bias as the DHS survey was conducted based on respondents’ self-reports. In addition, the temporal relationship between teenage pregnancy and explanatory variables couldn’t be established due to the cross-sectional nature of the survey.

## Conclusion

In the current study, one out of six young women aged 15 to 19 experienced teenage pregnancy. Being old, having a low level of education, being unmarried, working, having poor household wealth status, having late sexual initiation, utilizing contraceptives, being the head or spouse, having contraceptive knowledge, having an unmet need for contraception, and having high community contraceptive utilization were factors significantly associated with higher odds of teenage pregnancy. Therefore, addressing unmet needs for family planning, improving women’s educational status, and giving special attention to teenagers with low educational and economic status are recommended to reduce the prevalence and complications of teenage pregnancy. Further research is needed to explore the facilitators of teenage pregnancy prevention and focus on interventions to improve reproductive health among young women in sub-Saharan Africa. Policymakers can use this information to improve reproductive health programs and interventions and realize sustainable development goals by 2030 by investing in policy execution and appraisal, as well as engaging with stakeholders in teenagers’ reproductive and sexual health.

## Data Availability

No datasets were generated or analysed during the current study.

## Abbreviations

AOR: Adjusted Odds Ratio
CI: Confidence Interval
DHS: Demographic and Health Survey
ICC: Intra-class Correlation Coefficient
LMICs: Low- and middle-income countries
MOR: Median Odds Ratio
PCV: Proportional Change in Variance
SSA: Sub-Saharan Africa
VIF: Variance Inflation Factor
WHO: World Health Organization
